# Integration of LASER Diodes Emitting at Eight Different Wavelengths from Blue to Infrared on a 4H-SiC-Based Optical Integration Platform

**DOI:** 10.3390/ma19143145

**Published:** 2026-07-22

**Authors:** Xiaoshan Wang, Xiaoxuan Li, Ruyan Kang, Wenqi Jia, Xueyi Duan, Rongpeng Yang, Zhinuo Fan, Zechao Li, Jian Zhou, Zhiyuan Zuo

**Affiliations:** 1 Key Laboratory of Laser & Infrared System, Ministry of Education, Center for Optics Research and Engineering, Shandong University, Qingdao 266237, China; xs.wang@mail.sdu.edu.cn (X.W.); lixiaoxuan@mail.sdu.edu.cn (X.L.); 202212643@mail.sdu.edu.cn (X.D.);; 2 State Key Laboratory of Chips and Systems for Advanced Light Field Display, Shandong University-Hisense Optoelectronic Research Institute, Shandong University, Qingdao 266237, China202434140@mail.sdu.edu.cn (W.J.);; 3 East China Institute of Optoelectronic Integrated Device, Suzhou 215163, China; 4 Shandong Provincial Key Laboratory of Laser Technology and Application, Shandong University, Qingdao 266237, China

**Keywords:** 4H-SiC, laser integration, multi-wavelength, high-power, waveguide, photonic integration

## Abstract

We demonstrate an integrated eight-wavelength high-power laser source on a 4H-silicon carbide (SiC)-based optical integration platform. Eight discrete Fabry–Perot laser diodes emitting at 445 nm, 637 nm, 789 nm, 806 nm, 846 nm, 978 nm, 1316 nm, and 1552 nm are integrated on a single SiC chip, each delivering ≥100 mW continuous-wave output power. A complete fabrication process is developed, including lift-off metallization (Ni/Ti/Pt/Au), surface hydrophilic activation bonding, and multi-step blade dicing to form SiC waveguides with a width of 500 μm and a thickness defined by the ~510 μm dicing depth, matching the output aperture of the multimode laser diodes. The resulting waveguides exhibit a facet misorientation of <1° and an approximate facet mean surface roughness of ~2 nm. The laser diodes are directly butted against the waveguide facets for edge coupling, and fixed using In_52_Sn_48_ solder bonding with pulse temperature control. Under controlled temperature, all eight channels operate stably with measured peak wavelengths matching the design targets. This work provides a scalable and practical solution for multi-wavelength, high-power on-chip light source integration on the SiC platform, addressing critical thermal and integration challenges for dense wavelength division multiplexing.

## 1. Introduction

Silicon carbide (SiC) has emerged as a highly attractive platform for integrated photonics, owing to its unique combination of optical, thermal, and electronic properties [1,2,3,4]. 4H-SiC, in particular, offers a wide transparency window from near-ultraviolet to mid-infrared (0.35–5.5 μm), a large bandgap (3.2 eV) that suppresses two-photon absorption in the telecom band, and an exceptionally high thermal conductivity of 4.9 W/cm·K at 300 K—more than three times that of silicon [5,6,7,8,9]. Additionally, SiC features a high refractive index (~2.6 at 1550 nm) [10], enabling strong optical confinement, a reasonable nonlinear refractive index for four-wave mixing [11], and a significant second-order nonlinear susceptibility for second-harmonic generation and electro-optic modulation [12]. These properties, combined with mature wafer-scale fabrication processes inherited from the power electronics industry, make SiC a compelling candidate for next-generation photonic integrated circuits (PICs), particularly in applications demanding high power density, robust thermal management, and broadband operation [13,14].

The rapid growth of data center interconnects and high-bandwidth optical links has created an urgent need for multi-wavelength, high-power on-chip light sources [15,16,17]. Dense wavelength division multiplexing (DWDM) and high-density sensor fusion require a single chip to support multiple laser channels operating at distinct wavelengths, ranging from visible to near-infrared [18]. However, integrating such multi-channel laser arrays poses severe thermal challenges. For laser diodes, excessive heat accumulation leads to reduced output power, spectral instability, and accelerated aging. Moreover, conventional silicon-on-insulator (SOI) platforms suffer from the low thermal conductivity of the buried oxide layer (~1.4 W/m·K), which becomes a critical bottleneck for high-power integration. SiC, with its superior heat-spreading capability, offers a path to overcome this limitation [19]. It should be noted that the 500-μm-thick SiC layer used in this work is substantially thicker than the sub-micron layers typically employed in thin-film SiCOI photonics. Our structure is therefore better described as a large-cross-section SiC hybrid optical module, in which the thick SiC layer serves both as a mechanical support and as a multimode optical waveguide for high-power light transmission, rather than a conventional thin-film photonic integrated circuit.

Significant progress has been made in passive 4H-SiC-on-insulator photonics, including low-loss waveguides (~0.6 dB/cm) [20,21], micro-resonators [22,23], and optical parametric oscillators [24]. Nevertheless, all these demonstrations rely on external laser sources, and the integration of active light sources—especially multi-wavelength, high-power laser diodes—directly onto SiC platforms remains largely unexplored. A limited number of studies have reported III–V lasers bonded on SiC with improved thermal performance, but these works are restricted to single or few wavelengths (predominantly in the telecom band), fail to demonstrate on-chip coupling to SiC waveguides, and do not address simultaneous integration across the visible-to-infrared spectral range [25,26,27]. Unlike previous reports that rely on etching, the high-power multimode laser diodes (LDs) used in this work have a thickness exceeding 100 μm, which necessitates blade dicing rather than etching to form large-cross-section SiC waveguides.

To address these gaps, we present a complete process flow for a SiC-based integrated eight-wavelength light source. The eight wavelengths—445 nm, 637 nm, 789 nm, 806 nm, 846 nm, 978 nm, 1316 nm, and 1552 nm—are selected to cover key emission lines for visible-light communications, fluorescence sensing, and telecom/datacom applications, as well as to demonstrate the broadband capability of the SiC platform. Specifically, 445 nm and 637 nm are chosen for visible-light communications and fluorescence-based sensing; 789 nm, 806 nm, and 846 nm cover key wavelengths for solid-state laser pumping and atmospheric transmission windows for lidar applications; 978 nm serves as a standard pump wavelength for Yb-doped fiber lasers; and 1316 nm and 1552 nm correspond to the two principal low-loss transmission windows of optical fibers and the DWDM C-band. This deliberate selection allows the platform to demonstrate both application versatility and broadband optical capability. Each laser diode delivers an output power of at least 100 mW, and is directly butted against the input facet of a high-purity SiC waveguide for edge emission. The key challenges addressed include thermal management, precise multi-chip alignment, and the development of a full fabrication sequence comprising SiC thinning, SiO_2_ interlayer engineering, lift-off metallization, die bonding, wafer dicing, and end-face polishing. The fabricated waveguides exhibit good sidewall quality, which is essential for efficient light transmission. While direct propagation loss measurements were not performed in this study, the smooth cutting surface and the wide transparency window of 4H-SiC suggest favorable passive performance, which will be quantitatively characterized in future work. To the best of our knowledge, this is the first demonstration of an eight-wavelength, high-power, integrated laser source on a SiC platform with on-chip waveguide coupling.

## 2. Materials and Methods

**Figure 1:**
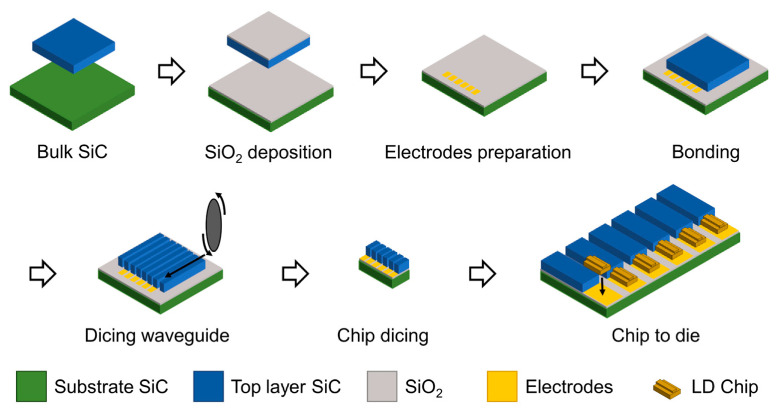
Process flowchart for the preparation of SiC on-chip integrated light source devices.

**Figure 2:**
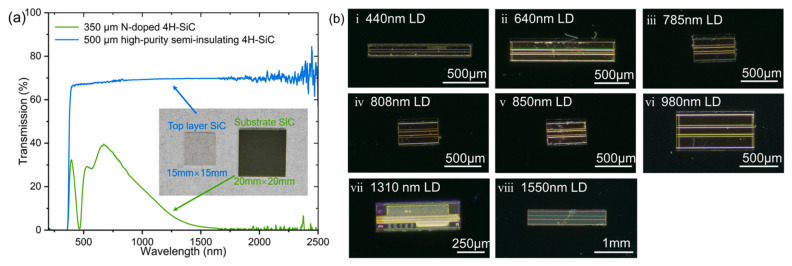
(**a**) Transmission spectra of the waveguide layer (semi-insulating) and substrate (n-type) SiC; (**b**) optical microscope images (OLYMPUS BX51, Tokyo, Japan, equipped with a CCD camera) of the eight high-power FP laser chips (i 440 nm, ii 640 nm, iii 785 nm, iv 808 nm, v 850 nm, vi 980 nm, vii 1310 nm, viii 1550 nm) used in this work.

In this work, two SiC substrates were used: one as a heat sink substrate in direct contact with the laser diodes (LDs), and the other as a waveguide layer, as shown in Figure 1. Compared with Si, SiC offers superior thermal conductivity, making it more suitable as a heat-spreading substrate. A 2.7 μm thick SiO_2_ layer was deposited on the n-type SiC heat sink substrate by high-density plasma chemical vapor deposition (HDP-CVD). Since light does not propagate in the substrate—owing to the presence of the SiO_2_ interlayer that prevents optical leakage—we chose a 350 μm thick n-type SiC to enhance thermal conductivity and reduce cost. Correspondingly, a 500 μm thick semi-insulating SiC was selected for the waveguide layer. The transmission curves of these two materials are shown in Figure 2a, where semi-insulating SiC has a higher transmittance from visible to near-infrared—about 70%. The absorption coefficient α can be estimated from the measured transmittance T and reflectance R using α = −(1/d) ln[*T*/(1 − *R*)], where d is the thickness. From our measurements, the absorption coefficient of SiC at 1550 nm is approximately 1.61 cm^−1^.

During operation, semiconductor laser diodes generate a large amount of heat. The accumulation of heat can limit the output capability of the laser, reduce output efficiency, and even cause device burnout. In this work, we use high-power multi-transverse-mode Fabry–Perot (FP) laser chips (i 440 nm, ii 640 nm, iii 785 nm, iv 808 nm, v 850 nm, vi 980 nm, vii 1310nm, viii 1550 nm), as shown in Figure 2b. These devices feature a large output aperture (chip thickness >100 μm) that supports multiple spatial modes, which is why we chose a large-waveguide cross-section (500 μm in width and ~510 μm in thickness) to maximize edge-coupling efficiency. While these FP lasers operate in multiple transverse modes, their longitudinal mode spacing is typically below the resolution of our spectrometers, resulting in the single-peak spectra. We provide the geometry parameters of the FP laser chips used in this work in the Table 1. And the LDs used in this work are commercial devices. The blue/green wavelength diodes (445 nm and 637 nm) are sourced from Juxin Optoelectronics (Shenzhen) Co., Ltd., a company based in Shenzhen, China, specializing in optical components and laser materials. The diodes at other wavelengths (789 nm, 806 nm, 846 nm, 978 nm, 1316 nm, and 1552 nm) are sourced from Sanan Optoelectronics in Xiamen, Fujian, China.

**Figure 3:**
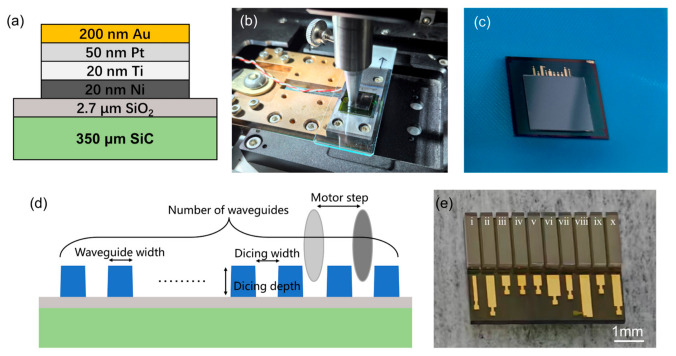
(**a**) Schematic diagram of preparing electrodes; (**b**) die bonding; (**c**) electrode-integrated SiC bonding substrate; (**d**) schematic diagram of SiC waveguide cutting scheme; (**e**) photograph of the light source device chip after slicing.

The electrodes were prepared using a lift-off process. Before metal deposition, the SiC heat sink substrate was cleaned, spin-coated with photoresist, pre-baked, aligned, exposed, and developed. After the development was completed, we cleaned the SiC heat sink substrate and inspected the photolithography pattern to complete the pre-inspection of metal film deposition. The deposition process used high-vacuum magnetron sputtering equipment (VJC-300, VNANO Co., Ltd., Melaka, Malaysia) to deposit 20 nm Ni/Ti layers in sequence, which are used as adhesion layers for electrodes. Next, deposit 50 nm Pt as an intermediate layer, which has excellent conductivity and oxidation resistance. It can also prevent diffusion between the Ti layer and the Au layer, avoid interface degradation, and thus improve the long-term reliability of the electrode. Finally, a 200 nm Au layer was deposited as the main electrode layer, as shown in Figure 3a. A thicker gold layer helps to improve the reliability and durability of the electrode. We used N-methyl-2-pyrrolidone (NMP) solvent to remove the photoresist at a process temperature of 50–60 °C. For thinner metal masks (less than the thickness of the photoresist), lift-off could be achieved within 30 s.

Furthermore, using surface hydrophilic activation bonding technology, we employed a submicron multi-function die bonder (GRD-TCB300S, Guanglinda Electronic Technology Co., Ltd., Suzhou, China) to complete the die bonding process of SiC chips, as shown in Figure 3b. The surface mount system mainly includes optical system, high-precision displacement control table, *Z*-axis pressure closed-loop system, a charge-coupled device (CCD) observation system, pulse temperature control system, inert gas protection system. We achieved high-precision alignment bonding between the SiC waveguide layer (15 mm × 15 mm) and the SiC heat sink substrate (20 mm × 20 mm) with an alignment accuracy within ±2 μm, as monitored by the CCD system during the bonding process. This bonding method offers the advantages of high bonding yield and large bonding area, providing a good foundation for subsequent dicing and waveguide preparation.

Due to the large output cross-section of the multimode LD, the waveguide cross-section must also be sufficiently large to maximize output power. The thickness of LD chips exceeds 100 μm, and the use of etching methods obviously cannot meet the requirements for fabricating of SiC waveguides thicker than 100 μm. At this size, SiC waveguides can be prepared by cutting. The cutting process method is shown in Figure 3d. In general, there are three main cutting methods: plasma cutting, laser cutting, and blade cutting. As an inert, hard, and brittle material, SiC is difficult to cut at thicknesses of several hundred microns using plasma or laser. Therefore, we mainly considered using mechanical processing methods, namely blade cutting. The cutting blade rotated at high speed, gradually cutting into the SiC wafer to form a waveguide structure. We therefore adopted a multi-step cutting scheme with four passes at depths of 150 μm, 150 μm, 110 μm, and 100 μm, respectively. The dicing width is defined by the cutting blade thickness of 260 μm, yielding an actual kerf width of approximately 265 μm as measured from cross-sectional optical microscope images. In addition, during the cutting process, it is necessary to use coolant to cool and lubricate the cutting area in order to reduce cutting heat and blade wear and improve cutting quality. After cutting, we placed the SiC bonding sheet in deionized water for ultrasonic cleaning to remove debris and impurities generated during the cutting process. The light source device chip after slicing, with dimensions of approximately 5 mm × 7 mm, is shown in Figure 3e.

**Figure 4:**
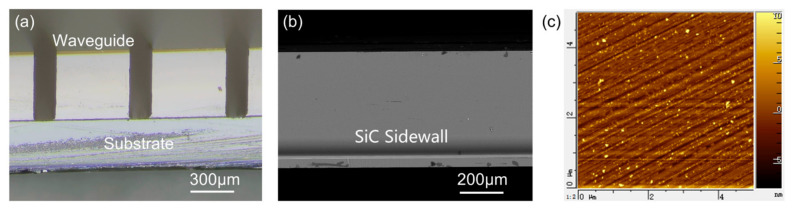
(**a**) Cross-sectional optical microscope image of the diced SiC waveguide; (**b**) SEM image of the SiC waveguide sidewall; (**c**) AFM image of the corresponding sidewall surface.

We characterized the surface morphology of the blade-cut waveguides. Cross-sectional optical microscope images are shown in Figure 4a,b. When the cutting depth reached approximately 510 μm, the facet misorientation was <1°, as measured from SEM images using image analysis software. Manual splitting was performed along the cutting groove, and the waveguide side section was characterized by scanning electron microscope (SEM). The sidewall surface was smooth and free of cracks. Furthermore, atomic force microscope (AFM) was used to characterize the surface roughness of the sidewall, as shown in Figure 4c. Within the measurement area of 5 μm × 5 μm, the facet mean surface roughness was approximately 2.0 nm (RMS), indicating that the SiC waveguides fabricated by the blade dicing method are of sufficient quality for the integration of SiC light source devices.

**Figure 5:**
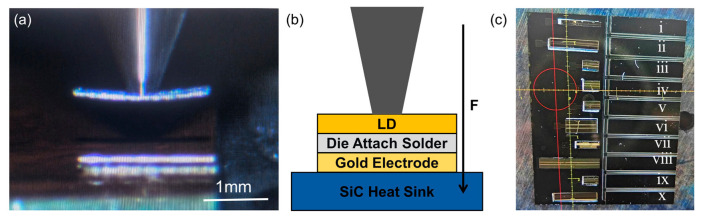
(**a**) Photo of the solder pads being aligned and placed. (**b**) Schematic diagram of interface forces during chip bonding. (**c**) Photo of the LD-integrated SiC light source device with eight active wavelength channels (positions i–viii, corresponding to 445 nm, 637 nm, 789 nm, 806 nm, 846 nm, 978 nm, 1316 nm, and 1552 nm, respectively) and two backup positions (ix and x).

We used In_52_Sn_48_ solder pads (25 μm thick) for die attachment, with the same planar dimensions as the LD chips. First, the solder preform was placed at the electrode position in Figure 5a. Then, an LD chip of the desired wavelength was placed onto the solder preform at the electrode position. Upon contact, the pressure sensor triggered a pre-set pulse temperature control program for heating and bonding as shown in Figure 5b. The temperature was ramped from room temperature to 150 °C at 5 °C/s, held for 30 s, and then cooled at 10 °C/s. After cooling, the suction nozzle was lifted manually, and the process was repeated for each LD chip to complete the multi-chip bonding in Figure 5c.

## 3. Results

**Figure 6:**
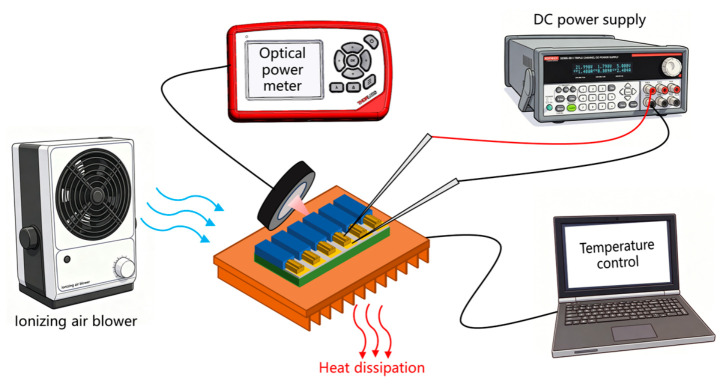
Schematic diagram of the test setup for the SiC integrated light-source device.

To characterize the light-source devices and ensure stable high-performance operation, appropriate testing and characterization methods are crucial. Figure 6 shows the testing device for a custom-built SiC integrated light source device, which mainly includes a homemade chip constant temperature stage, a DC power supply (30V, 3A, 2231A-30-3, Keithley, Solon, OH, USA), a manual probe stage, a temperature control system, and a CCD observation system. The testing of power and spectrum for SiC light source devices requires some peripheral equipment, such as ionizing air blower, collimators, optical power meters, spectrometers, etc. The performance, reliability, and lifetime of semiconductor lasers strongly depend on operating temperature stability. The performance, reliability, and lifetime of semiconductor lasers strongly depend on operating temperature stability. Our preliminary experiments revealed that excessive temperature or fluctuations cause severe adverse effects: thermal stress can lead to failure or detachment of the LD bonding interfaces; device aging accelerates, output power drops, threshold current increases, and noise rises. Moreover, under high-current driving, heat accumulates inside the chip, and thermal runaway may completely destroy the laser. Therefore, temperature control and heat dissipation are crucial for semiconductor lasers. The LD temperature was controlled by a constant-temperature stage with a proportional–integral–derivative (PID) system, which maintains the stage temperature within ±0.05 °C of the set point.

**Figure 7:**
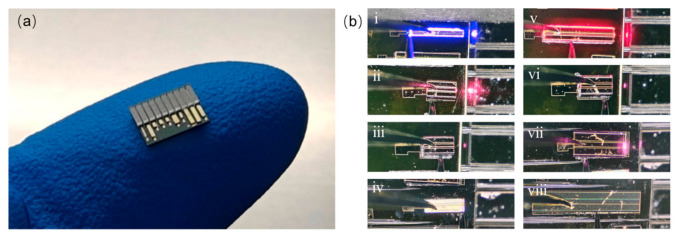
(**a**) Photo of the complete eight-wavelength LD-integrated SiC integrated light source device. (**b**) Photos of the optical emission from each LD during testing (positions i–viii corresponding to 445, 637, 789, 806, 846, 978, 1316, and 1552 nm, respectively).

The SiC light source device prepared through a series of processing steps is shown in Figure 7a. After the device was installed on the self-assembled constant-temperature stage, we turned on the refrigeration system, set the target temperature to 15 °C, and waited for the system to stabilize. For each wavelength channel, electrical contact was established using a manual probe station under CCD observation: the positive probe contacted the top electrode of the LD, and the negative probe contacted the common substrate electrode. The light output after power-on is shown in Figure 7b. The integrated chip contains eight semiconductor lasers with the following wavelengths (440 nm, 640 nm, 785 nm, 980 nm, 808 nm, 850 nm, 1310 nm and 1550 nm). The two extra channels designed in the figure are backup channels, serving as backup positions for LD chips that may fail during fabrication or testing.

**Figure 8:**
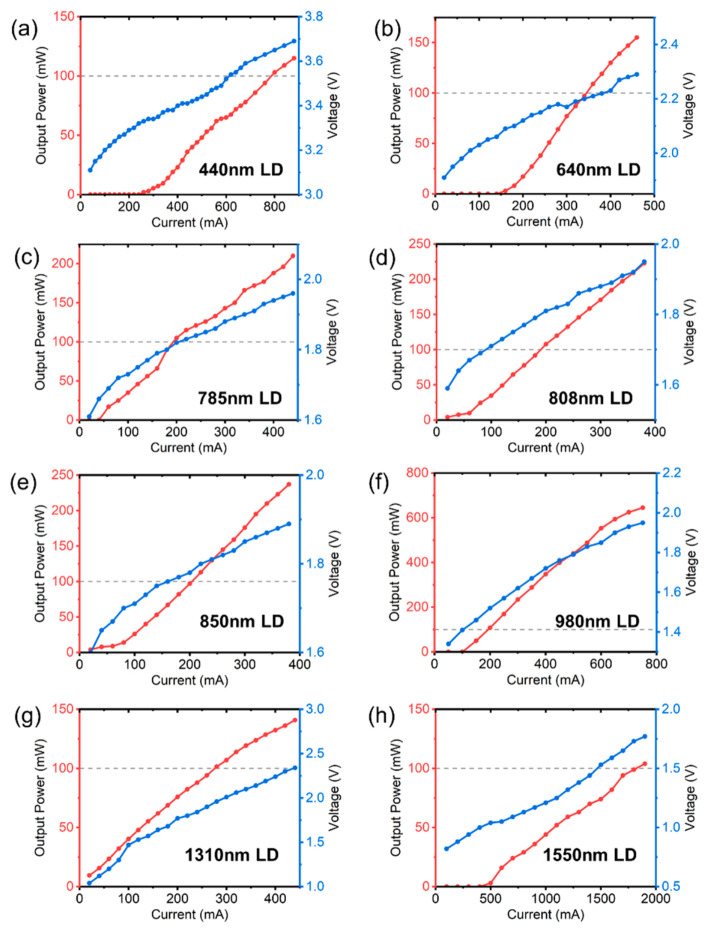
Output power characteristics of the eight-wavelength semiconductor lasers on the SiC light source device: (**a**) 440 nm, (**b**) 640 nm, (**c**) 785 nm, (**d**) 808 nm, (**e**) 850 nm, (**f**) 980 nm, (**g**) 1310 nm, and (**h**) 1550 nm wavelength semiconductor lasers.

The measurement procedure consisted of two stages. In the first stage, the output power was measured. The optical power meter probe was aligned with the output facet of the corresponding SiC waveguide to collect all emitted light. The current source was then turned on, and the driving current was increased in 20-mA steps. After each step stabilized, the driving current, forward voltage, and output optical power were recorded until the preset voltage limit was reached. A complete P-I-V curve was thus obtained, from which the maximum output power within the safe driving range was determined as shown in Figure 8.

In addition to the power measurements, we characterized the spectral linewidth of each LD channel at its maximum output power. The full width at half maximum (FWHM) values for channels 1–8 (corresponding to 445 nm, 637 nm, 789 nm, 806 nm, 846 nm, 978 nm, 1316 nm, and 1552 nm) are 3.7 nm, 2.6 nm, 4.0 nm, 4.22 nm, 4.5 nm, 4.8 nm, 2.2 nm, and 4.3 nm, respectively. These values are consistent with the typical spectral characteristics of multimode Fabry–Perot laser diodes operating under continuous-wave conditions, where the longitudinal mode spacing is generally below the spectrometer resolution, resulting in an envelope spectrum with the reported FWHM. The measured peak wavelengths and corresponding FWHM values are summarized in Table 2. The optical power measured at the SiC waveguide output facet represents the net transmitted power after edge coupling from the LD, propagation through the ~4.5 mm long diced waveguide, and output extraction from the facet. While a full optical budget—including coupling efficiency, propagation loss, and insertion loss—was not measured in this study, the measured output power exceeding 100 mW per channel confirms the viability of the integration approach and provides a foundation for future quantitative characterization.

**Figure 9:**
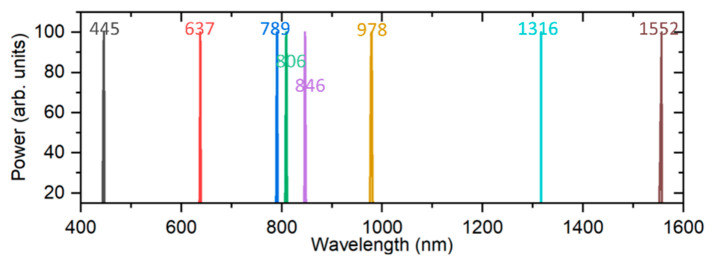
Peak wavelengths of each channel output from the eight-wavelength SiC integrated light source device.

In the second stage, the output wavelength at the peak power was measured. After the power test, the current was reset to zero, the power meter was removed, and appropriate optical paths were configured for different wavelength bands. For the 1310 nm and 1550 nm channels, a multiwavelength spectrometer (AQ6370E, Yokogawa, Tokyo, Japan) with a lensed fiber was used. For the visible and near-infrared channels, the spectrometer input (PG2000pro, Fosun Optics, Shanghai, China) was directly aligned with the waveguide. The driving current was increased to the maximum value determined in the power test, stable spectra were acquired, and the peak wavelength was recorded. Finally, the measured peak wavelengths of the eight channels were 445, 637, 789, 806,846, 978, 1316, and 1552 nm (Figure 9).

## 4. Conclusions

In this work, we demonstrate an integrated eight-wavelength high-power laser source on a 4H-SiC-on-insulator platform. Eight discrete Fabry–Perot laser diodes emitting at 445 nm, 637 nm, 789 nm, 806 nm, 846 nm, 978 nm, 1316 nm, and 1552 nm are integrated on a single SiC chip. A complete fabrication process is developed, including lift-off metallization (Ni/Ti/Pt/Au), hydrophilic activation bonding, and multi-step blade dicing to form large-cross-section SiC waveguides. The resulting waveguides exhibit a facet mis-orientation of <1° and an approximate facet mean surface roughness of ~2 nm over a 5 μm × 5 μm area. The laser diodes are directly butted against the waveguide facets for edge coupling and fixed using In_52_Sn_48_ solder bonding with pulse temperature control, achieving a high-precision alignment process. Under temperature control, the peak output power of all eight wavelengths exceeds 100 mW. The waveguides exhibit smooth sidewalls and high-quality facets, which are favorable for low-loss light transmission. Quantitative characterization of propagation loss and coupling efficiency will be addressed in future work. Unlike previous III-V/SiC demonstrations restricted to single or few telecom wavelengths, our work extends the integration to eight wavelengths from visible to near-infrared. Nevertheless, the measured output power has not been de-embedded into coupling, propagation, and extraction losses; the large waveguide cross-section is not suitable for dense photonic integration; and all channels were tested individually without simultaneous operation or reliability assessment. Quantitative characterization of propagation loss and coupling efficiency, along with simultaneous multi-channel testing, will be addressed in future work. This work establishes a scalable process for multi-wavelength, high-power on-chip light source integration on the 4H-SiC-based optical integration platform, paving the way for dense wavelength division multiplexing and broadband sensing applications that demand high power density and reliable thermal management.

## Figures and Tables

**Table 1:** Specifications of the high-power FP lasers used in the experiments.

LD Wavelength (nm)	Length (μm)	Width (μm)	Height (μm)	Rib Width (μm)
440	1200	150	110	30
640	1500	340	130	80
785	500	320	120	75
808	500	320	120	75
850	500	320	120	75
980	1000	520	125	100
1310	750	250	130	65
1550	2000	400	105	100

**Table 2:** Test parameters for each channel of 8-channel LD-integrated SiC integrated light source device.

Chip Channel	Threshold Current (mA)	Threshold Voltage (V)	Slope Efficiency (W/A)	Maximum Output Power (mW)	FWHM (nm)
i 440 nm	240	3.32	0.18	119	3.7
ii 640 nm	140	2.05	0.47	153	2.6
iii 785 nm	80	1.70	0.55	206	4.0
iv 808 nm	45	1.58	0.64	223	4.2
v 850 nm	20	1.60	0.72	235	4.5
vi 980 nm	60	1.36	1.01	610	4.8
vii 1310 nm	20	1.04	0.29	140	2.2
viii 1550 nm	350	1.01	0.08	114	4.3

## Data Availability

The original contributions presented in this study are included in the article. Further inquiries can be directed to the corresponding authors.

## Abbreviations

The following abbreviations are used in this manuscript:

4H-SiCOI	4H-silicon carbide-on-insulator
AFM	atomic force microscope
CCD	charge-coupled device
DWDM	dense wavelength division multiplexing
FP	Fabry–Perot
HDP-CVD	high-density plasma chemical vapor deposition
LDs	laser diodes
NMP	N-methyl-2-pyrrolidone
PICs	photonic integrated circuits
PID	proportional–integral–derivative
RMS	root mean square
SEM	scanning electron microscope
SOI	silicon-on-insulator
